# Different Profiles of Antibodies and Cytokines Were Found Between Severe and Moderate COVID-19 Patients

**DOI:** 10.3389/fimmu.2021.723585

**Published:** 2021-08-19

**Authors:** Yaolin Guo, Tianyi Li, Xinyi Xia, Bin Su, Hanping Li, Yingmei Feng, Jingwan Han, Xiaolin Wang, Lei Jia, Zuoyi Bao, Jingyun Li, Yongjian Liu, Lin Li

**Affiliations:** ^1^Department of AIDS Research, State Key Laboratory of Pathogen and Biosecurity, Beijing Institute of Microbiology and Epidemiology, Beijing, China; ^2^Institute of Laboratory Medicine, Jinling Hospital, Nanjing University School of Medicine, The First School of Clinical Medicine, Southern Medical University, Nanjing, China; ^3^Beijing Youan Hospital, Capital Medical University, Beijing, China

**Keywords:** SARS-CoV-2, COVID-19, neutralizing antibody, immunoglobulin type, cytokine

## Abstract

**Objectives:**

Our objective was to determine the antibody and cytokine profiles in different COVID-19 patients.

**Methods:**

COVID-19 patients with different clinical classifications were enrolled in this study. The level of IgG antibodies, IgA, IgM, IgE, and IgG subclasses targeting N and S proteins were tested using ELISA. Neutralizing antibody titers were determined by using a toxin neutralization assay (TNA) with live SARS-CoV-2. The concentrations of 8 cytokines, including IL-2, IL-4, IL-6, IL-10, CCL2, CXCL10, IFN-γ, and TNF-α, were measured using the Protein Sample Ella-Simple ELISA system. The differences in antibodies and cytokines between severe and moderate patients were compared by t-tests or Mann-Whitney tests.

**Results:**

A total of 79 COVID-19 patients, including 49 moderate patients and 30 severe patients, were enrolled. Compared with those in moderate patients, neutralizing antibody and IgG-S antibody titers in severe patients were significantly higher. The concentration of IgG-N antibody was significantly higher than that of IgG-S antibody in COVID-19 patients. There was a significant difference in the distribution of IgG subclass antibodies between moderate patients and severe patients. The positive ratio of anti-S protein IgG3 is significantly more than anti-N protein IgG3, while the anti-S protein IgG4 positive rate is significantly less than the anti-N protein IgG4 positive rate. IL-2 was lower in COVID-19 patients than in healthy individuals, while IL-4, IL-6, CCL2, IFN-γ, and TNF-α were higher in COVID-19 patients than in healthy individuals. IL-6 was significantly higher in severe patients than in moderate patients. The antibody level of anti-S protein was positively correlated with the titer of neutralizing antibody, but there was no relationship between cytokines and neutralizing antibody.

**Conclusions:**

Our findings show the severe COVID-19 patients’ antibody levels were stronger than those of moderate patients, and a cytokine storm is associated with COVID-19 severity. There was a difference in immunoglobulin type between anti-S protein antibodies and anti-N protein antibodies in COVID-19 patients. And clarified the value of the profile in critical prevention.

## Introduction

Severe acute respiratory syndrome coronavirus 2 (SARS-CoV-2) has spread worldwide for more than a year. As of March 2021, SARS-CoV-2 has infected more than 100 million people worldwide, resulting in more than 2 million deaths, and the global epidemic situation of SARS-CoV-2 remains serious. Individuals infected by SARS-CoV-2 have different clinical symptoms. Most individuals have moderate symptoms, such as fever, respiratory tract symptoms, and imaging features of pneumonia. Approximately 14% of people have severe symptoms (1), such as respiratory distress and respiratory rate ≥30 times/min, mean oxygen saturation ≤93% at resting-state, or arterial blood oxygen partial pressure (PaO2)/oxygen concentration (FiO2) ≤300 mmHg (1 mmHg = 0.133 kPa) and progressive aggravation of clinical symptoms. Considering that the case-fatality rate of critical COVID-19 patients is as high as 2.3% (1–3), it is important to clarify the internal mechanism of severe illness.

Antibodies, especially neutralizing antibodies, are associated with the severity of the patient (4, 5). According to the difference of serum antibody content, the evaluation of antibody level mainly focused on the titer of IgA, IgM and IgG. Most individuals diagnosed with SARS-CoV-2 infection by PCR will produce IgA, IgM, and IgG against the spike protein (S) and nucleocapsid protein (N) within 1-2 weeks of symptoms, and remain elevated following initial viral clearance (6–11). Previous studies ignored that the highest content of IgG in serum has four different functional subclasses, only one study on IgG subtypes has been reported (12). IgE is extremely scarce, but IgE is related to hypersensitivity, which has been less studied in SARS-CoV-2 (13). Also, neutralizing antibodies (nAbs) can bind to viral particles and prevent them from entering host cells, and provide specific immune defense for infected patients. Thus, the level of neutralizing antibodies can be used to judge the ability of the body to resist the virus. Researchers have already shown that neutralizing antibodies that can block virus infection have a good therapeutic effect on patients with COVID-19 (14–19). There is evidence that neutralizing antibodies to SARS-CoV-2 infection persist for months, and a higher titer of nAbs, IgG, and IgM is independently associated with a worse clinical classification (20–25).

Aggressive inflammatory response and the release of a large amount of pro-inflammatory cytokines, or cytokine storm has been reported to be involved in COVID-19 severe pathogenesis (26, 27). IL-2 can stimulate the proliferation of NK cells and secrete a variety of cytokines, increase the cytotoxicity (28, 29). IL-4 can specifically induce Th2 cells and stimulate the proliferation of activated B cells and T cells (30–33). IL-6 is a major highly inducible pro-inflammatory cytokine, induces IL-8 and MCP-1 secrete, increases vascular permeability during the early phase of inflammation (34–36). IL-10 is a key anti-inflammatory mediator ensuring protection of a host from over-exuberant responses to pathogens and microbiota (37–39). CCL2 and CXCL10 are chemokines, CCL2 facilitates the migration and infiltration of monocytes/macrophages to sites of inflammation produced by either tissue injury or infection, CXCL10 could drive longer duration of mechanical ventilation during COVID-19 ARDS (40–42). TNF-a and IFN-γ mediated inflammatory cell death signaling pathways to limiting tissue damage in COVID-19 patients (43). An increasing amount of clinical data suggests that a cytokine storm is associated with COVID-19 severity and is also a crucial cause of death from COVID-19, including IL-2, IL-6, IL-7, IL-10, G-CSF, IP-10, MCP-1, MIP-1α, and TNF-α (44–49).

However, the number of cases in previous studies is small, and no comprehensive and systematic study has been performed. In this study, we intend to collect a cross-sectional sample of moderate and severe patients to systematically analyze the distribution of anti-N protein and anti-S protein IgA, IgM, IgE, IgG1, IgG2, IgG3, and IgG4 and the relationship among IgG antibody against spike protein (IgG-S), nucleoprotein (IgG-N), nAbs and 8 cytokines (IL-2, IL-4, IL-6, IL-10, CCL2, CXCL10, IFN-γ, and TNF-α). The immunological differences between moderate and severe patients were compared to clarify the value of the profile in critical prevention.

## Methods

### Patients and Data Sources

This study included 79 patients with COVID-19 and 10 healthy controls. According to the Chinese Government Diagnosis and Treatment Guideline (8^th^ edition), the clinical classification was divided into moderate patients and severe patients. To inactivate the serum and plasma sample complement, all samples were incubated in a water bath at 56°C for 30 minutes.

### IgG Antibody of Spike Protein and Nucleoprotein Measurement

In our study, the materials for indirect chemical luminescence analysis (CLIA) to measure IgG antibodies against SARS-CoV-2 spike protein and nucleoprotein were provided by Beijing KEWEI Clinical Diagnostic Reagent Inc. Unlike traditional enzyme-linked reaction kits, the CLIA method can quickly and effectively quantify antibody content. To detect IgG, 100 μL of sample diluent was added to each 96-well plate, and 10 μL of serum, plasma or standard S0-S6 was added to the corresponding well and incubated at 37°C for 15 minutes. We then washed the plate five times with 300 μL of wash buffer. Then, 100 μL conjugate dilution was added to each well and incubated at 37°C for 15 minutes. After the second wash cycle, 50 μL glowing substrates A and 50 μL glowing substrates B were added. After mixing, the solution was incubated in the dark at room temperature for 1-5 minutes. Then, the plate was placed on an ELISA reader to acquire a relative light unit (RLU). The specific content of the specimen IgG antibody can be calculated by the standard curve from the standard S0-S6.

### Toxin Neutralization Assay (TNA) With Live SARS-CoV-2

The neutralization titer was determined in Vero E6 cells, and the live strain SARS-CoV-2 (BetaCoV/Beijing/AMMS01/2020) used in the neutralization assay was isolated from a throat swab of a COVID-19 patient (50). To achieve 90-95% confluence of Vero E6 cells, a 96-well plate with 1×10^4^ Vero E6 cells per well was incubated at 37°C and 5% CO_2_ one day in advance. The live strain SARS-CoV-2 of 120 TCID_50_ was mixed with inactivated serum and plasma that had been serially diluted 2-fold, from 1:10 to 1:1280, and incubated for 1 h at 37°C and 5% CO_2_. Each concentration had two repetitions. Then, the mixture was added to 96-well plates of Vero cells and cultivated at 37°C and 5% CO_2_ with daily microscopic examination for cytopathic effects (CPEs). On day 3, the titers of antibody were calculated as the reciprocal of the highest dilution at which the CPE was completely inhibited on the well (51). If no neutralization reaction was observed in the patient sample at the initial dilution concentration of 1:10, we regarded the neutralization antibody titer as 0.

### Typing of Anti-S Protein and Anti-N Protein Antibodies

ELISA plates, coated with the SARS-CoV-2 N protein or S protein in advance, were used to detect the classification of immunoglobulins in moderate patients and severe patients. First, 100 μL sample diluent was added to each well, and 10 μL 1:20 diluted serum and plasma or standard from the kit was added to the corresponding well and incubated at 37°C for 60 minutes. Then, the plate was washed five times with 300 μL of wash buffer. One of seven antibodies (including HRP-monoclonal mouse anti-human IgG1, IgG2, IgG3, IgG4, IgA, IgM, and IgE) diluted 1:1000 was added to 100 μL in each well and incubated at 37°C for 30 minutes. After the second wash cycle, 50 μL glowing substrate A and 50 μL glowing substrate B were added. After incubating for 15 minutes in the dark, 50 μL stop solution was added to each well. Then, the plate was placed on an ELISA reader to detect the absorbance at 450 nm and 630 nm. The cutoff value was calculated, and samples with a fluorescence value greater than 0.2 were regarded as positive; otherwise, they were regarded as negative.

### Cytokine Measurement

The concentrations of 8 cytokines, including IL-2, IL-4, IL-6, IL-10, CCL2, CXCL10, IFN-γ, and TNF-α, were measured using the Protein Sample Ella-Simple ELISA system (Revision 1.1, Mar 2020) following the manufacturer’s instructions. The sera of healthy individuals (n=10) were included as controls.

### Statistical Analysis

All continuous variable descriptions are described as medians (IQRs), and categorical characteristics are described as numbers (%). Means for continuous variables were compared using independent group t-tests when the data were normally distributed; otherwise, the Mann-Whitney test was used. All statistical analyses were performed using SPSS (Statistical Package for the Social Sciences) version 21.0 software (SPSS Inc), and a *P*-value <0.05 was considered statistically significant.

## Results

### Demographic Characteristics

The demographic characteristics of these patients are summarized in **Table 1**. This study included 49 moderate patients and 30 severe patients. There were 45 females and 34 males among a total of 79 COVID-19 patients. The median age of the study’s patients was 63.4 years, and 70.89% of patients were more than 60 years old. We enumerated the PCR positive detection time of moderate patients and severe patients, and found that there was no difference in PCR positive detection time between the two groups by the Mann-Whitney test. Average ages of moderate and severe patients were similar, at 63.6 and 63, respectively. Of all enrolled patients, 59.49% had complications. Among the 47 patients with complications, hypertension, diabetes, and heart-related disease accounted for the largest proportion, at 44.68%, 36.17%, and 19.15%, respectively. Moreover, patients with a history of disease accounted for 65.96% and 34.04% of moderate and severe patients, respectively.

**Table 1:** Baseline characteristics of patients infected with COVID-19.

Characteristic	Moderate Patients	Severe Patients	Total
	(n = 49)	(n = 30)	(n = 79)
Sex			
Female	30	15	45
Male	19	15	34
Age, y			
35-39	2	2	4
40-49	3	0	3
50-59	10	6	16
60-69	20	16	36
70-79	11	4	15
80-89	3	2	5
Median (IQR)	63.6	63	63.4 (58–69)
Weeks after PCR positivity			
1	6	3	9
2	11	4	15
3	2	7	9
4	14	5	19
5	12	8	20
6	3	1	4
7	1	2	3
Comorbidities			
Hypertension	13	8	21
Diabetes	11	6	17
Heart-related disease	7	2	9

### Neutralizing Antibody Titers in Moderate and Severe COVID-19 Patients

**Figure 1:**
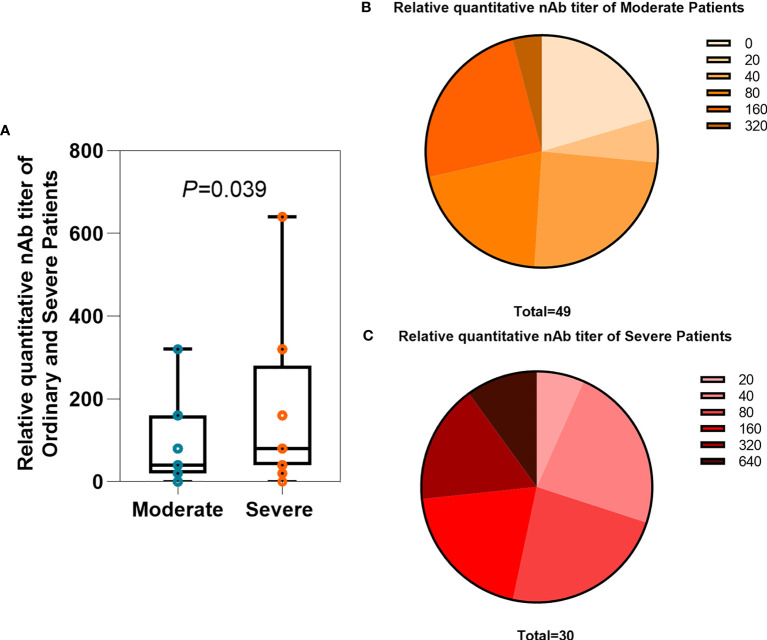
Comparison of relative quantitative nAb titers between the moderate and severe groups. **(A)** Average titers of neutralizing antibodies in moderate (n = 49) and severe (n = 30) patients. The box plots show the medians (middle line) and first and third quartiles (boxes), and the whiskers show the minimum and maximum. T-test *P*-values are depicted in the plots. **(B, C)** The percentage of moderate and severe patients with neutralizing antibody titers.

A total of 79 serum and plasma samples in all groups of patients were analyzed for the titers of neutralizing antibodies with TNA. The clinical laboratory ELISA setup results in discrete titers of 0, 20, 40, 80, 160, 320, or 640. The neutralizing antibody titer in severe patients was significantly higher than that in moderate patients, with geometric mean reciprocal titers of 79.59 and 164.4, respectively (**Figure 1A**, *P*=0.039). According to the study described by Wajnberg et al. (52), titers of 20, 40, 80, and 160 were categorized as low titers, 320 as moderate, and 640 as high titers. For plasma therapy, titers of 320 or higher were initially deemed eligible. Of the 49 moderate patients, 10 (20.41%) had a titer of 0, 3 (6.12%) of 20, 12 (24.49%) of 40, 10 (20.41%) of 80, 12 (24.49%) of 160, and 2 (4.08%) of 320 (**Figure 1B**). Of the 30 severe patients, 2 (6.67%) of 20, 7 (23.33%) of 40, 7 (23.33%) of 80, 6 (20%) of 160, 5 (16.67%) of 320, and 3 (10%) of 640 (**Figure 1C**). Thus, the vast majority of patients (87.34. %) with COVID-19 have neutralizing antibodies. However, only a small number of patients (12.66%) reached a qualified plasma therapeutic titer greater than or equal to 320.

### IgG-N and IgG-S Antibody Levels in Moderate and Severe COVID-19 Patients

**Figure 2:**
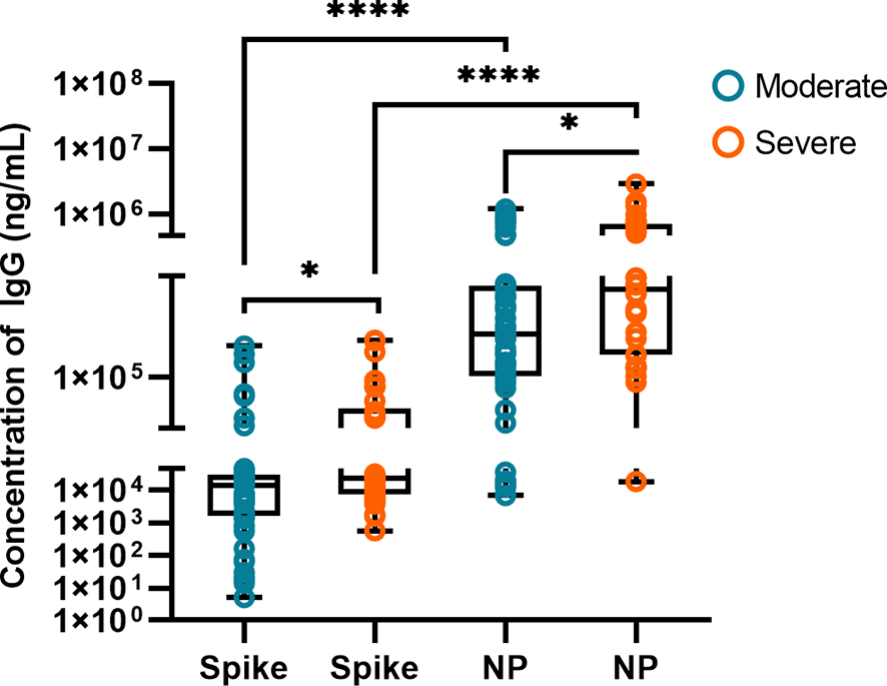
Comparison of IgG-S and IgG-N antibodies in the moderate (n = 49) and severe (n = 30) groups. The box plots show the medians (middle line) and first and third quartiles (boxes), and the whiskers show the minimum and maximum. Mann-Whitney test *P*-values are depicted in the plots. *p < 0.05, ****p < 0.0001.

We quantitatively detected IgG antibody concentrations of IgG-S and IgG-N to explore the two major antibodies in patients with COVID-19 against SARS-CoV-2. **Figure 2** shows the comparison of spike protein and nucleoprotein IgG in the moderate (n=49) and severe (n=30) groups. The median values of IgG-S concentration in severe patients (22748 ng/mL) were significantly higher than in moderate patients (14403 ng/mL, *P*<0.05). Moderate patients with IgG-S below the median had shorter PCR positivity time than those with IgG-S above the median (*P*<0.05). The positivity time of PCR in severe patients with IgG-S lower than the median was shorter than that with higher median (*P*<0.01). IgG-N in severe patients was significantly higher than in moderate patients, with median values of 380249 ng/mL and 191578 ng/mL, respectively (*P*<0.05). There was no difference in PCR positivity time between moderate and severe patients when IgG-N was below or above the median (*P*>0.05). The median values of IgG-N concentration were 13.30 times higher than IgG-S in moderate patients and 16.72 times higher than IgG-S in severe patients (P<0.0001). We stratified moderate and severe COVID-19 patients by PCR positive detection time, and the difference of IgG-S and IgG-N concentrations is shown in **Figure S1**.

### Relationship Between IgG-N, IgG-S, and Neutralizing Antibodies

**Figure 3:**
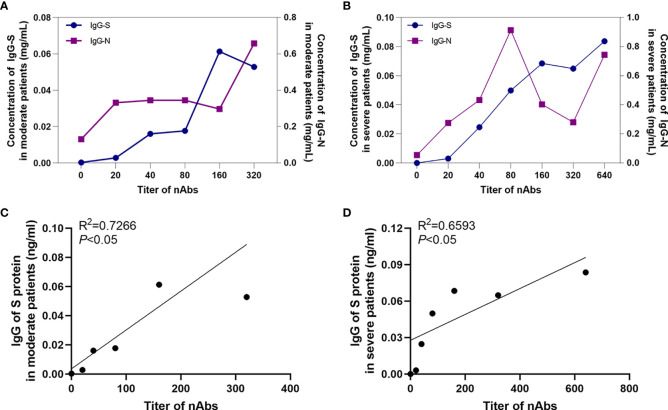
The correlation between antibodies. The relationship between IgG-S and IgG-N antibody levels and nAb titers in moderate patients **(A)** and severe patients **(B)**. The correlation between IgG-S antibody levels and nAb titers in moderate patients **(C)** and severe patients **(D)**.

Correlation analysis suggested that the IgG-S antibody concentration increased with increasing neutralizing antibody titer in both moderate and severe patients (**Figure 3**). Analysis showed that there was a positive correlation between IgG-S antibodies and neutralizing antibodies in moderate and severe COVID-19 patients (*P*<0.05), and the regression equations were Y=0.0002665*X+0.00357 and Y=0.0001065* X+0.02778, respectively.

### Classification of Anti-S Protein and Anti-N Protein Antibodies

**Figure 4:**
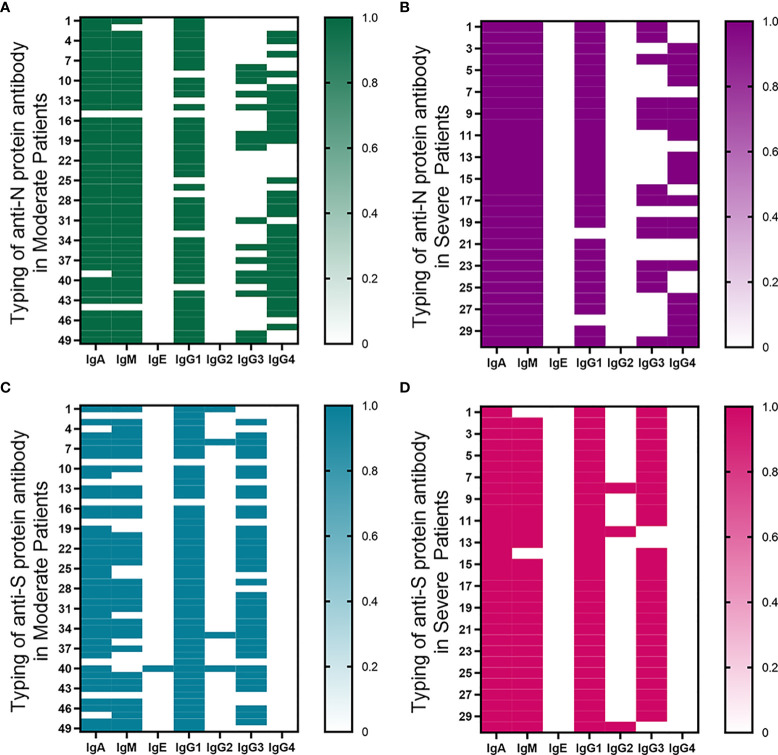
Classification of anti-S protein and anti-N protein antibodies. **(A, B)** The typing of anti-N protein immunoglobulin in moderate and severe patients. **(C, D)** The typing of anti-S protein immunoglobulin in moderate and severe patients. Samples with a fluorescence value greater than 0.2 were regarded as positive, with an assignment of 1; otherwise, they were regarded as negative, with an assignment of 0.

In moderate and severe COVID-19 patients, the positive ratio of anti-N protein antibodies was IgA≥IgM>IgG, and the positive ratio of anti-S protein antibodies was IgG≥IgA>IgM (**Figure 4**). IgA of anti-S protein and anti-N protein was positive in severe patients, while the detection time of IgA-S and IgA-N negative was shorter than that of PCR positive in moderate patients (*P*<0.05). IgM of anti-N protein was positive in all severe patients, but IgM of anti-S protein was negative in only two severe patients. Only the detection time of IgM-N negative was shorter than that of PCR positive in moderate patients (*P*<0.05). Almost no positive IgE was detected.

IgG1 is the most abundant IgG subclass of anti-N protein and anti-S protein antibodies, while IgG2 is the least abundant IgG subclass. The positive ratio of anti-S protein IgG3 was significantly higher than that of anti-N protein IgG3, while the anti-S protein IgG4 positive rate was significantly lower than the anti-N protein IgG4 positive rate.

### Cytokine Concentrations

**Figure 5:**
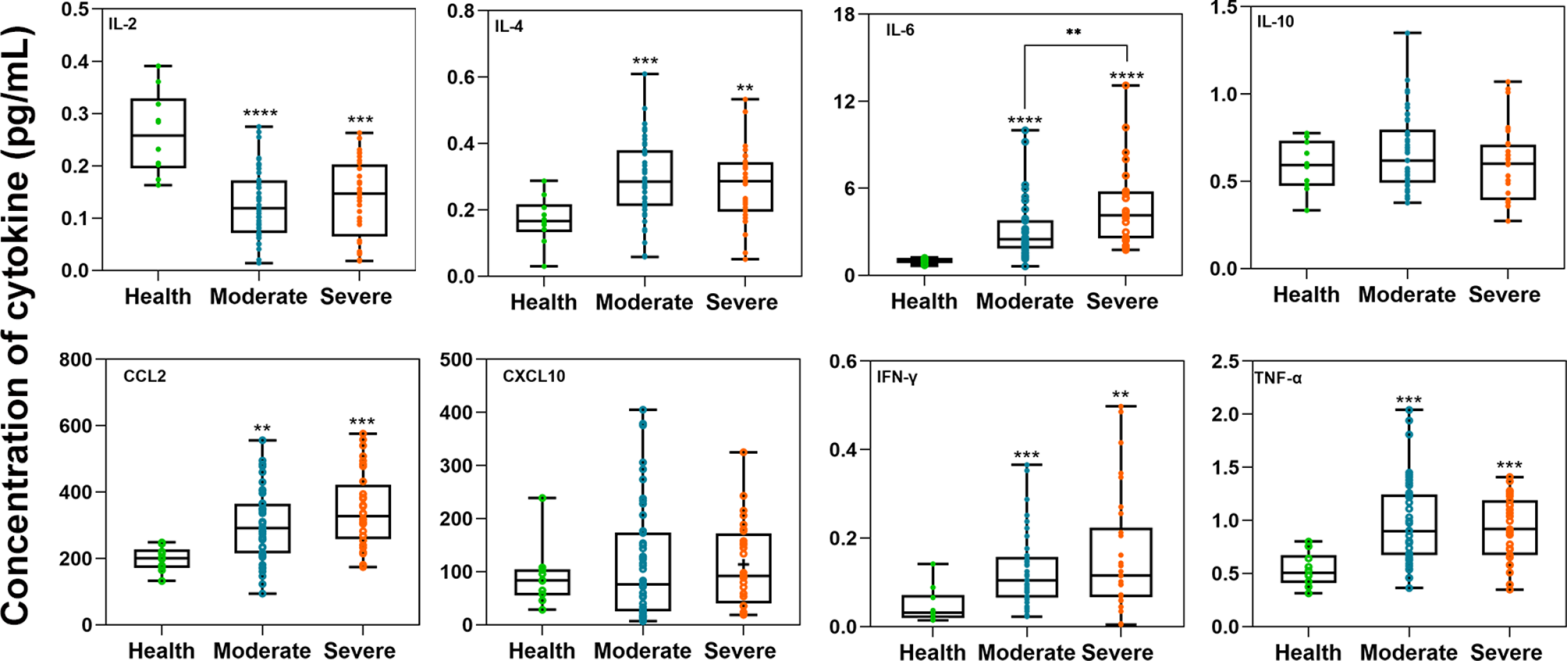
The comparison of cytokine concentrations between the healthy donor and moderate or severe groups. The unit of the eight cytokine concentrations was pg/mL. The box plots show the medians (middle line) and first and third quartiles (boxes), and the whiskers show the minimum and maximum. Mann-Whitney test *P*-values are depicted in the plots. I bars indicate standard deviations. **p < 0.01, ***p < 0.001, ****p < 0.0001.

We included ten healthy donors as controls to compare the concentrations of IL-2, IL-4, IL-6, IL-10, CCL2, CXCL10, IFN-γ, and TNF-α between moderate and severe patients in **Figure 5**. Through the Mann-Whitney test, we found that only the average concentrations of IL-2 were higher in healthy controls than in COVID-19 patients (*P*<0.001). In both moderate and severe COVID-19 patients, the average concentrations of IL-4, IL-6, IL-10, CCL2, IFN-γ, and TNF-α (*P*<0.05) were higher than those in healthy donors. Among them, IL-6 in severe patients was significantly higher than that in moderate patients. There was no relationship between cytokines and neutralizing antibodies, as determined by Spearman analysis.

## Discussion

In this study, we characterized the antibody and cytokine responses in COVID-19 patients. The proportion of patients with a history of disease in moderate and severe patients is more than 50%, which may accelerate the disease process of patients with COVID-19 (53, 54).

The average neutralizing antibody titer and IgG-S of severe patients were significantly higher than those of moderate patients. Neutralizing antibodies can block viral infection and have a good therapeutic effect on patients with COVID-19 (14–18). Recent research found that higher viral loads and stronger antibody responses are related to more severe disease status in patients with SARS-CoV-2 infection (21, 55). These results suggest that patients with more severe clinical typing may have a stronger immunity to the virus (56). It is worth noting that the later stage of severe patients is not caused by SARS-CoV-2 but may be caused by their own immune damage.

From our quantitative data, we confirmed the conclusion of previous studies that PCR positive time is related to antibody concentration (57), and we found that the IgG-N concentration was higher than the IgG-S concentration (*P*<0.0001), not only because the nucleoprotein released by SARS-CoV-2 is more abundant than the spike protein but also because the spike protein has structural changes between prefusion and postfusion (58). Most of the antibodies produced by the innate immune system target the postfusion spike protein, which causes the body to overreact and produce an antibody-dependent enhancement effect (ADE) (59). In addition, most of the immune responses activated by SARS-CoV-2-infected humans produce IgG-N with no neutralizing activity in the early phase after symptom onset (60), which is also difficult in the development of neutralizing antibodies and vaccines. Additionally, we compared the neutralizing antibodies, IgG-N antibodies, and IgG-S antibodies of COVID-19 patients with different clinical symptoms. There is a positive correlation between the nAbs and IgG-S antibodies, due to the existence of sites on the S protein that bind to multiple receptors of target cells (61–63).

The positive detection rate of IgA and IgM in COVID-19 infection was related to the positive detection time of PCR and Clinical classification. In the early stage of infection, with the increase of infection time, the detection rate of antibody was higher (22, 64, 65). The positive rates of anti-N protein IgA and IgM antibodies were higher than anti-S protein, which was similarly related to the structure of SARS-CoV-2 mentioned above. Secretory IgA (SIgA) is the main component of the mucosal defense system. The content of SIgA in respiratory secretions directly affects the resistance of respiratory mucosa to pathogens, and there is a positive correlation between them (66). Although the role of serum IgA is relatively unexplored, it can transmit activating signals, leading to phagocytosis, respiratory burst, ADCC, increased antigen presentation, degranulation and cytokine release (67–69). Upregulated IgA production may be the result of increased levels of TGF-β and IL-10, which promote antibody switching in SARS-CoV-2 infection (70, 71).

The first line of defense for severe patients to prevent pathogens from invading the body is stronger. Among anti-N and anti-S protein antibodies, IgG1 is the most common IgG subclass, and the anti-S protein IgG3 positive rate is significantly higher than the anti-N protein IgG3 positive rate. IgG3 and IgG1 exhibit the most efficient activation of the classical complement cascade, but IgG4 generally cannot activate this cascade (72). Therefore, the S protein of SARS-CoV-2 is more likely to trigger effector functions, including complement activation, antibody-mediated phagocytosis, or Ab-mediated cellular cytotoxicity (ADCC) (73). The N protein of SARS-CoV-2 is a chronic antigen that stimulates the production of most terminal IgG4 subclasses (72).

Six cytokines in COVID-19 patients show differences from those in healthy individuals, but they were not related to the antibody titer. IFN-γ, TNF-α, and IL-2 are the main inflammatory factors of Th1 cytokines and can induce pathogenesis. Similar to previous research, IFN-γ and TNF-α are significantly higher in COVID-19 patients than in healthy individuals. However, in contrast to previous studies, our data show that the concentration of IL-2 in COVID-19 patients is significantly lower than that in healthy individuals (48, 74–77). This difference may be related to sample sources and clinical treatment, which suggests that cytokine storms are different in different populations even infected with the same pathogen. Th2 cytokines, including IL-4, IL-6, and IL-10, have anti-inflammatory effects and can prevent pathogenesis and alleviate diseases. IL-4, as a part of the cytokine storm associated with severe respiratory symptoms, is significantly higher in COVID-19 patients than in healthy individuals (74, 78). Our study found that IL-6 is related not only to the severity of SARS symptoms but also to SARS-CoV-2 infection (79, 80). The proinflammatory chemokine CCL2 can mediate the directional migration of immune cells and is also higher in COVID-19 patients than in healthy individuals (81). The data show that severe clinical symptoms are closely related to the joint action of a variety of cytokines (44, 82–84).

## Conclusion

In summary, the nAbs and IgG-S in severe patients were significantly higher than those in moderate patients, and the two antibodies had a positive correlation. There was a difference in immunoglobulin type between anti-S protein antibody and anti-N protein antibody in COVID-19 patients. Anti-S protein IgG3 was significantly more abundant than anti-N protein IgG3, while anti-N protein IgG4 was significantly more abundant than anti-S IgG4. Many cytokines in COVID-19 patients were significantly different from those in healthy individuals, including IL-2, IL-4, IL-6, CCL2, IFN-γ, and TNF-α. There was no relationship between cytokines and neutralizing antibodies. The different immune characteristics of COVID-19 patients with different clinical types were significant for the systematic study of the pathogenic mechanism of SARS-CoV-2.

## Data Availability Statement

The original contributions presented in the study are included in the article/**Supplementary Material**. Further inquiries can be directed to the corresponding authors.

## Ethics Statement

The studies involving human participants were reviewed and approved by Beijing Youan Hospital Research Ethics Committee (No. 2020-037). The patients/participants provided their written informed consent to participate in this study.

## Author Contributions

TL, YL, and LL contributed to conception and design of the study. XX, BS, and YF contributed to sample collection. YG contributed to organized the database, performed the statistical analysis, wrote the first draft of the manuscript. HL, JH, and XW contributed to bibliographic survey. LJ, ZB, and JL were involved in the critical revision of the article. All authors contributed to the article and approved the submitted version.

## Funding

Supported by the key special project of “Technology for Economy 2020” of Beijing Municipal Commission of Science and Technology, the Emergency Key Program of Guangzhou Laboratory (Grant No. EKPG21-01), the National Natural Science Foundation of China (81772165, 81974303 to BS), and the China Primary Health Care Foundation-Youan Medical Development Fund (BJYAYY-2020PY-01 to BS).

## Conflict of Interest

The authors declare that the research was conducted in the absence of any commercial or financial relationships that could be construed as a potential conflict of interest.

## Publisher’s Note

All claims expressed in this article are solely those of the authors and do not necessarily represent those of their affiliated organizations, or those of the publisher, the editors and the reviewers. Any product that may be evaluated in this article, or claim that may be made by its manufacturer, is not guaranteed or endorsed by the publisher.
