# Supplementary material for: Different Profiles of Antibodies and Cytokines Were Found Between Severe and Moderate COVID-19 Patients
Source: Front Immunol. 2021 Aug 19;12:723585. doi: 10.3389/fimmu.2021.723585 (PMC8417126; doi:10.3389/fimmu.2021.723585)
Supplement: Supplementary file 1 [file DataSheet_1.docx]

**
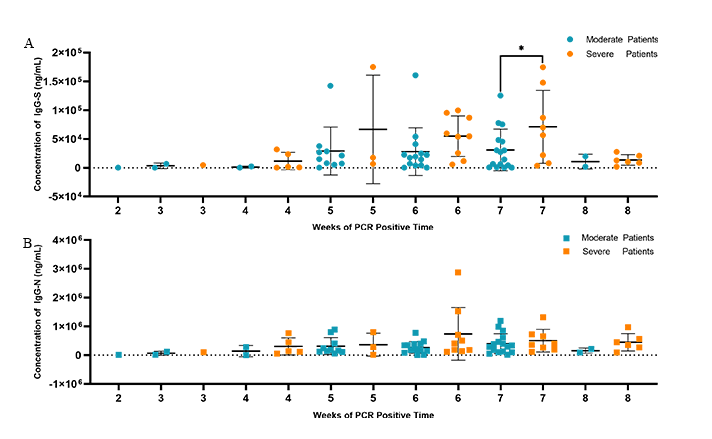
SUPPLEMENTARY FIGURES**

**Figure S1.** The relationship between PCR positive time with IgG-S (A) and IgG-N (B) antibodies in the moderate (n=49) and severe (n=30) groups. Mann-Whitney test *P*-values are depicted in the plots. I bars indicate standard deviations
